# Multi-Target Tracking Using Windowed Fourier Single-Pixel Imaging

**DOI:** 10.3390/s21237934

**Published:** 2021-11-28

**Authors:** Jinyu Zhang, Taiyang Hu, Xiaolang Shao, Mengxuan Xiao, Yingjiao Rong, Zelong Xiao

**Affiliations:** 1 School of Electronic Engineering and Optical Technology, Nanjing University of Science and Technology, Nanjing 210094, China; zhangjinyu@njust.edu.cn (J.Z.); xl_shao1994@126.com (X.S.); MengxuanXiao@njust.edu.cn (M.X.); zelongxiao@njust.edu.cn (Z.X.); 2 Science and Technology on Near-Surface Detection Laboratory, Wuxi 214035, China; enjoy_rong@163.com

**Keywords:** single-pixel imaging, multi-target tracking, windowed Fourier transform, fast moving object tracking

## Abstract

The single-pixel imaging (SPI) technique enables the tracking of moving targets at a high frame rate. However, when extended to the problem of multi-target tracking, there is no effective solution using SPI yet. Thus, a multi-target tracking method using windowed Fourier single-pixel imaging (WFSI) is proposed in this paper. The WFSI technique uses a series of windowed Fourier basis patterns to illuminate the target. This method can estimate the displacements of *K* independently moving targets by implementing 6K measurements and calculating 2K windowed Fourier coefficients, which is a measurement method with low redundancy. To enhance the capability of the proposed method, we propose a joint estimation approach for multi-target displacement, which solves the problem where different targets in close proximity cannot be distinguished. Using the independent and joint estimation approaches, multi-target tracking can be implemented with WFSI. The accuracy of the proposed multi-target tracking method is verified by numerical simulation to be less than 2 pixels. The tracking effectiveness is analyzed by a video experiment. This method provides, for the first time, an effective idea of multi-target tracking using SPI.

## 1. Introduction

Multi-target detection and tracking have widespread applications in military defense [1,2], area surveillance [3,4], UAV detection [5,6], and other fields. Compared with single target tracking, multi-target tracking is more widely used and more difficult to realize. There are two types of conventional multi-target tracking methods. The first one is an image-free method, such as laser radar and millimeter radar, which emits electromagnetic waves with different beam steering to illuminate the target and receives the back-scattered echoes to estimate the target’s position and velocity. The multi-target tracking capability of the radar relies on the construction of multiple beams, and the antenna system is more difficult to design. Moreover the tracking systems usually require multi-channel compounding that is complex and costly. The other one is an image-based method, which estimates the displacements of the moving objects by acquiring successive reconstructed images and analyzes the trajectory of the moving object from a few frames [7,8]. The block matching method is a typical multi-target tracking method [9,10]. Compared to radar systems, image-based target tracking systems are less expensive. However, high-speed cameras are costly, and the data throughput is usually huge in a short amount of time. In addition, high-speed imaging devices in the millimeter waveband are more challenging to be designed.

With the development of optical digital modulating devices (DMDs) and millimeter-wave metasurface materials, the single-pixel imaging (SPI) technique, which can be used to develop an imaging system, has attracted the attention of many scholars in recent years [11,12,13,14,15,16]. The SPI technique uses different modulated patterns to illuminate the target and recovers the target image by a computational method. It can be applied to develop imaging systems with a low system complexity in, e.g., millimeter and terahertz wavebands [17,18,19,20]. Due to the flexible and broadband system composition of SPI, it is also promising for the detection and tracking of moving objects. In addition, the accuracy of an SPI imaging system is affected by the motion blur of the target. It has been shown that, if the target motion parameter can be estimated accurately, it can also be of great value for the imaging calibration of moving targets [14]. The studies on the use of SPI for imaging and tracking of moving targets are as follows.

Jiao et al. proposed a motion estimation and image quality enhancement scheme for a single reconstructed image [21]. The optimal motion parameters and reconstructed images can be searched based on the connection between the image reconstruction variance and the accuracy of the estimated motion parameters. By performing motion estimation between subsequent frames, another estimation method proposed by S. Monin et al. may be applied to any type of trajectory [22]. Their estimation methods based on one or a few image frames are time-consuming. Moreover, the moving target estimated by their methods is a single target.

The measurement data used for imaging is often redundant for the estimation of motion parameters. From this perspective, scholars have tried to reduce the number of measurements to increase the frame rate of target tracking. Shi et al. proposed a method to obtain the position of the object in the scene from the 1D projected curves [23]. This method demonstrates that reducing redundancy in data acquisition is an effective approach for the fast tracking method. In [24,25], the method proposed by Zhang et al., based on Fourier single-pixel imaging (FSI), uses six Fourier basis patterns for structured light modulation to measure only two Fourier coefficients to estimate the displacement of a moving object. Recently, a faster method was proposed by Zha and Shi et al.; it requires only three geometric moment patterns to illuminate a moving object in one frame [26]. These works fully demonstrate that the trajectory of a moving target in a scene can be estimated with low redundancy using a small amount of measurement data. However, the problem of tracking multiple targets remains unsolved.

Thus, a multi-target tracking method using windowed Fourier single-pixel imaging (WFSI) is proposed in this paper. Inspired by the successful application of the short-time Fourier transform (STFT) in signal processing, we designed a series of window functions that vary with the target positions. The WFSI technique uses windowed Fourier basis patterns to illuminate the target. Combining these different window functions, the problem of tracking multiple targets can be transformed into the problem of tracking a single target. One window function is designed for each target, and two windowed Fourier coefficients are calculated by six measurements to complete the estimation of the displacement of this target. For the *K* target, 6K measurements are required. In addition, considering the scene that two targets are close to each other, a multi-target joint estimation approach is proposed to enhance the capability of the proposed tracking method. In addition, a joint multi-target estimation approach is proposed to enhance the capability of the proposed tracking method in consideration of the two targets in close proximity. Numerical simulation experiments are conducted to verify the multi-target tracking capability of the proposed method. We also analyzed the effectiveness of multi-target tracking through a video experiment. Consequently, the proposed method enables multi-target tracking in real-time and for a long duration.

## 2. Principle and Method

### 2.1. Single-Target Tracking Method Using FSI

The FSI technique can reconstruct high-quality images by acquiring their Fourier spectrum [27]. The tracking of a single target can be estimated by the FSI technique by measuring 2 Fourier coefficients [24]. FSI employs a series of Fourier basis patterns to illuminate the target, and each Fourier basis pattern is characterized by its spatial frequency pair $\left(f_{x},f_{y}\right)$ and its initial phase ϕ:

(1) $$P\left(x,y,\phi \right)=A+B\cdot cos\left(2\pi f_{x}x+2\pi f_{y}y+\phi \right)$$

where (x,y) denotes 2-D coordinates in the spatial domain, *A* is the average intensity of the pattern, and *B* denotes the contrast. The Fourier basis patterns can be converted from grayscale to binary by employing image upsampling and the Floyed–Steinberg error diffusion dithering method [28]. Using the binary Fourier basis pattern to illuminate the target image I(x,y), the total light intensity D is equivalent to an inner product of the target image I(x,y) and the binary Fourier basis pattern $P_{bin}\left(x,y\right)$:

(2) $$D\left(f_{x},f_{y},\phi \right)=\;\int \int \;I\left(x,y\right)P_{bin}\left(x,y,\phi \right)dxdy$$

The Fourier coefficients of the target image can be acquired using the three-step phase-shifting algorithm [24]:

(3) $$F_{I}\left(f_{x},f_{y}\right)=\left[2D\left(f_{x},f_{y},0\right)-D\left(f_{x},f_{y},\frac{2\pi }{3}\right)-D\left(f_{x},f_{y},\frac{4\pi }{4}\right)\right]+\sqrt{3}j\cdot \left[D\left(f_{x},f_{y},\frac{2\pi }{3}\right)-D\left(f_{x},f_{y},\frac{4\pi }{3}\right)\right]$$

The integral in Equations (2) and (3) implies that the Fourier transform is a global-to-point transformation. The change of the target’s position in the spatial domain leads to the change of the entire Fourier coefficients in the spatial frequency domain. Therefore, the presence of a target in space can be determined by detecting one or a few Fourier coefficients in the spatial frequency domain.

The scene image containing a moving target can be represented as the sum of the target image and the background image:

(4) $$I_{scene}\left(x,y;t\right)=I_{target}\left(x,y;t\right)+I_{background}\left(x,y\right)$$

The Fourier spectrum of the target image is equal to the difference between the Fourier spectrum of the scene image and the background image, so background subtraction can be applied to obtain the Fourier spectrum of the target:

(5) $$\begin{array}{cc} F\{I_{target}\left(x,y;t\right)\} & =F\{I_{scene}\left(x,y;t\right)-I_{background}\left(x,y\right)\}\\ \; & =F\left\{I_{scene}\left(x,y;t\right)\right\}-F\left\{I_{background}\left(x,y\right)\right\} \end{array}$$

where F{} denotes the Fourier transform. For the sake of convenience of expression, if not specifically stated, I(x,y) denotes the target image after background subtraction. For a moving target, the displacement (Δx,Δy) from $t_{1}$ to $t_{2}$ in the spatial domain results in a phase shift in the spatial frequency domain:

(6) $$I\left(x,y;t=t_{2}\right)=I\left(x+\Delta x,y+\Delta y;t=t_{1}\right)$$

(7) $$F_{I}\left(f_{x},f_{y};t=t_{2}\right)=F_{I}\left(f_{x},f_{y};t=t_{1}\right)\cdot e^{-j2\pi (f_{x}\Delta x+f_{y}\Delta y)}$$

where $F_{I}\left(f_{x},f_{y}\right)$ denotes the Fourier coefficients of the image I(x,y). According to Equation (12), it can be found that the displacement of the target (Δx,Δy) can be calculated from the phase term $\Delta \varphi =-j2\pi (f_{x}\Delta x+f_{y}\Delta y)$. In order to solve for Δx and Δy separately, a system of equations needs to be established using at least two different pairs of spatial frequency $\left(f_{x},f_{y}\right)$. For simplicity, the spatial frequency pairs, $\left(f_{x},0\right)$ and $\left(0,f_{y}\right)$ can be used. Therefore, the displacement (Δx,Δy) can be derived through the following:

(8) $$\Delta x=-\frac{1}{2\pi f_{x}}\cdot arg\left\{\frac{F_{I}\left(f_{x},0;t=t_{2}\right)}{F_{I}\left(f_{x},0;t=t_{1}\right)}\right\}$$

(9) $$\Delta y=-\frac{1}{2\pi f_{y}}\cdot arg\left\{\frac{F_{I}\left(0,f_{y};t=t_{2}\right)}{F_{I}\left(0,f_{y};t=t_{1}\right)}\right\}$$

where arg{} denotes the argument operation.

### 2.2. Multi-Target Tracking Method Using WFSI

It was demonstrated that only 2 Fourier coefficients measurements (6 Fourier basis patterns) are needed to achieve the detection and tracking of a moving target, in the above-mentioned section. However, when there are multiple moving targets in the scene, it is incapable of distinguishing different targets in the spatial frequency domain due to the global-to-point property of the Fourier transform.

Inspired by the STFT, we tried to design different window functions in the spatial domain to transform the multi-target tracking into single-target tracking. If there are *K* targets, the scene I(x,y) in Equation (2) needs to be modified as the sum of multi-target images $I_{i}\left(x,y\right)$:

(10) $$I\left(x,y;t\right)=\sum_{i=1}^{K}I_{i}\left(x,y;t\right)$$

The spatial window functions $w_{i}\left(x,y;t\right)$ are designed to satisfy the following:

(11) $$I_{i}\left(x,y;t\right)=I\left(x,y;t\right)\cdot w_{i}\left(x,y;t\right)$$

**Figure 1:**
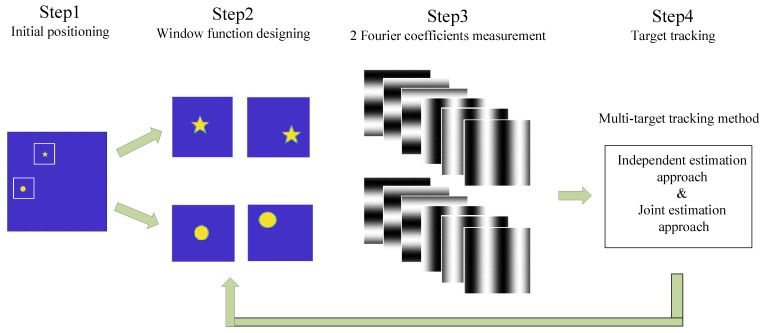
Sketch of multi-target tracking using WFSI. The process is as follows: Firstly, locate the initial multi-target positions. Secondly, design the window functions according to the positions. Thirdly, implement 6K measurements to calculate 2K Fourier coefficients. After that, using the multi-target tracking method with the independent approach and joint approach, the displacements can be estimated. Then, according to the estimated result, redesign the window functions to continue multi-target tracking.

Based on the WFSI technique, firstly we design appropriate window functions to transform the multi-target tracking problem into a single-target tracking problem. Afterwards, 6*K* measurements are implemented to calculate 2*K* Fourier coefficients. After that, using the multi-target tracking method including the independent approach and joint approach, the displacements of the multi-target can be estimated. Lastly, according to the estimated result, the window functions are redesigned to continue the multi-target tracking. The sketch of multi-target tracking using WFSI is shown in Figure 1.

**Figure 2:**
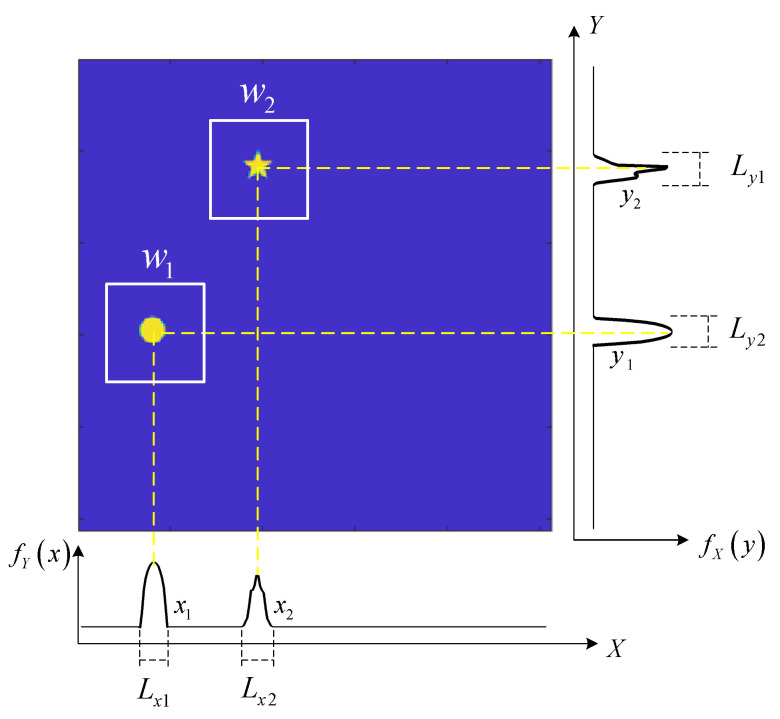
Illustration of multi-target locating via the projected curves method. $f_{Y}\left(x\right)$ and $f_{X}\left(y\right)$ are the projection of the scene image on the *X* axis and the *Y* axis. $\left(x_{1},x_{2}\right)$ and $\left(y_{1},y_{2}\right)$ are the regions where targets exist on the *X* axis and the *Y* axis. $\left(L_{x1},L_{x2},L_{y1},L_{y2}\right)$ are the length of the regions. $\left(w_{1},w_{2}\right)$ represents the window functions designed for the two targets.

Two moving targets are illustrated as an example, as shown in Figure 2. The window function is binary that can be regular or special in shape. In this paper, a rectangular window function is selected, the center of the rectangular window is the target center location, and the window length is taken as four times the target size. There are many methods to determine the approximate area of the target center position. We applied the projection curve method proposed in [23] to determine the positions of the targets. This projection curve method constructs modulation information that satisfies the projection conditions and can transform 2D images into 1D projection curves. The 1D projection curves, which provide the location information of the moving object, can be obtained with a high resolution in real time. In this paper, the scene image is projected on the *X* axis and *Y* axis, respectively. For an image with 512×512 pixels, a compressed sensing method is used to select 2×0.3×512 patterns to obtain the two projection curves. From these two curves, it is easily determined that there are four approximate regions for these two targets {$\left(x_{1},y_{1}\right)$, $\left(x_{2},y_{2}\right)$, $\left(x_{1},y_{2}\right)$, and $\left(x_{2},y_{1}\right)$ }. Among these four regions, targets existed in only two of them. The Fourier coefficients for the image of regions where no targets exist are approximately zero. For *K* moving targets, using the projected curve method, less than $K^{2}$ possible regions are obtained. Therefore, the multi-target position can be determined according to the 2K Fourier coefficients of the target image after weighting the *K* window functions. In fact, only one Fourier coefficient is needed to determine the presence of a target in the region. Here, we chose 2 Fourier coefficients because the later tracking methods require 2 Fourier coefficients. It should be noted that the proposed method cannot distinguish between two targets if their spatial locations overlap with each other.

### 2.3. Independent Estimation Approach

After designing the spatial window functions, the Fourier coefficients of each target image $I_{i}\left(x,y\right)$ can be denoted as

(12) $$F_{I_{i}}\left(f_{x},f_{y};t=t_{2}\right)=F_{I_{i}}\left(f_{x},f_{y};t=t_{1}\right)\cdot e^{-j2\pi (f_{x}\Delta x_{i}+f_{y}\Delta y_{i})}$$

where $\left(\Delta x_{i},\Delta y_{i}\right)$ denote the displacements of each target from $t_{1}$ to $t_{2}$. According to Equations (2) and (11), the light intensity $D_{i}\left(f_{x},f_{y},\phi \right)$ is the integral value of the inner product of the entire image I(x,y), the spatial window function $w_{i}\left(x,y\right)$, and the binary Fourier basis pattern $P_{bin}\left(x,y\right)$. The sampling matrix designed by the DMD device is the product of $w_{i}\left(x,y\right)$ and $P_{bin}\left(x,y\right)$. The WFSI technique uses windowed Fourier basis patterns to illuminate the target, which can be expressed as Equation (13).

(13) $$D_{i}\left(f_{x},f_{y},\phi \right)=\;\int \int \;I\left(x,y\right)\cdot w_{i}\left(x,y\right)\cdot P_{bin}\left(x,y,\phi \right)dxdy$$

The windowed Fourier coefficients of each target $I_{i}\left(x,y\right)$ can be calculated by applying the three-step phase-shifting algorithm according to Equation (3). Thus, the displacement $\left(\Delta x_{i},\Delta y_{i}\right)$ of each target can be obtained by calculating the phase term $\Delta \varphi_{i}=-j2\pi \left(f_{x}\Delta x_{i}+f_{y}\Delta y_{i}\right)$ according to Equation (12).

**Figure 3:**
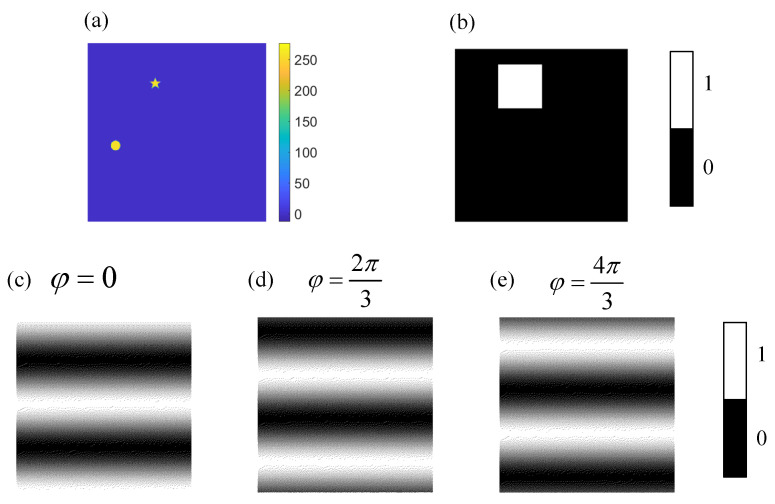
Illustrations of the measurement process. (**a**) is the target image with 256×256 pixels, (**b**) is the designed window function with 256×256 pixels. (**c**–**e**) are the binary Fourier patterns $P_{bin}\left(\frac{2}{N},0,\varphi \right)$ with 512×512 pixels used to calculate the Fourier coefficient $F_{I2}\left(\frac{2}{N},0\right)$.

The window function $w_{2}\left(x,y\right)$ in Figure 2 is shown again in Figure 3b. The 3 binary Fourier patterns ($P_{bin}\left(\frac{2}{N},0,0\right)$, $P_{bin}\left(\frac{2}{N},0,\frac{2\pi }{3}\right)$, $P_{bin}\left(\frac{2}{N},0,\frac{4\pi }{3}\right)$) used to calculate the Fourier coefficient $F_{I_{2}}\left(\frac{2}{N},0\right)$ are shown in Figure 3c–e.

The multi-target tracking method described above is limited to an assumption that the target moves within the region of the spatial window function, but this assumption is not easily satisfied over a long duration. Updating the center positions of the window functions corresponding to different targets in real time during the measuring process is one useful approach. Therefore, a strategy to dynamically alter the window position is used in this paper. The center of the *i*th window function $\bar{w_{i}}$ changes according to the estimation result of the displacement $\left(\Delta x_{i},\Delta y_{i}\right)$ of the *i*th target each time, which is denoted as

(14) $$\begin{array}{cc} {\bar{w_{i}}}^{\left(k+1\right)} & =(x_{i}^{\left(k\right)},y_{i}^{\left(k\right)})\\ \; & =(x_{i}^{\left(k-1\right)}+\Delta x_{i}^{\left(k-1\right)},y_{i}^{\left(k-1\right)}+\Delta y_{i}^{\left(k-1\right)}) \end{array}$$

where the superscript *k* represents the *k*th measurement.

In this way, if the displacement of a target is estimated accurately and the window length is bigger than the displacement between 2 frames, the target will always be within the window. Therefore, the upper limit of the target velocity that can be estimated by the proposed method is determined by the selected window length. The window length should not be too large. An excessively long window length can lead to overlapping window functions, and the estimated result will produce errors.

### 2.4. Joint Estimation Approach

When the distance between two targets in the scene is less than the length of the designed rectangular window, the multi-target tracking cannot be transformed into single-target tracking. At this point, the choice of window shape becomes very important if we want to distinguish two targets by the window function. However, the designing of special-shaped windows is a more difficult problem. Thus, a joint estimation approach is proposed to solve the problem of target discrimination when different targets are within one window. Due to the linear feature of the Fourier transform, the Fourier spectrum of *M* targets within a spatial region W(x,y) is equal to the sum of the Fourier spectrum of *M* single target $T_{i}\left(i=1,2,...,M\right)$, which can be denoted as

(15) $$F\left\{I\left(x,y\right)W\left(x,y\right)\right\}=F\left\{\sum_{T_{i}\in W}I_{i}\left(x,y\right)\right\}$$

which means

(16) $$\begin{array}{cc} F_{IW}\left(f_{x},f_{y};t=t_{2}\right) & =\sum_{T_{i}\in W}F_{I_{i}}\left(f_{x},f_{y};t=t_{2}\right)\\ \; & =\sum_{T_{i}\in W}F_{I_{i}}\left(f_{x},f_{y};t=t_{1}\right)\cdot e^{-j2\pi (f_{x}\Delta x_{i}+f_{y}\Delta y_{i})} \end{array}$$

In the case where the Fourier spectrum of every target has been determined, there are 2M unknown variables in Equation (16). Therefore, the displacements $\left(\Delta x_{i},\Delta y_{i}\right)$ of these *M* targets can be calculated by solving a system of equations corresponding to 2M different spatial frequencies $\left(f_{x},f_{y}\right)$. Equation (16) is a nonlinear system of equations, and the computational difficulty of calculating the roots increases rapidly with the increase of *M*. There are two types of methods for solving nonlinear equations: the analytical method and the iterative search method. The analytical method is faster than the search method and it is generally accepted that nonlinear equations have an analytic solution when M<5. Considering the solution complexity and real-time requirement, the joint estimation of more than 5 targets is hard to achieve. In this paper, we use the analytical method to solve 2 targets within one window.

## 3. Results

### 3.1. Simulation

**Figure 4:**
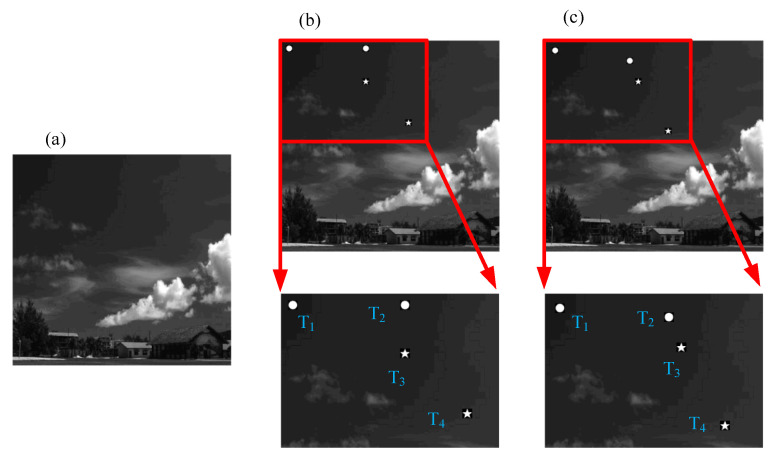
Simulation scenes: (**a**) a complex background, (**b**) a scene image at moment $t_{1}$, and (**c**) a scene image at moment $t_{2}$. There are four moving targets in the scene, denoted as $T_{1},T_{2},T_{3},T_{4}$.

Numerical simulations are employed to study the proposed method. The scene for the simulation is shown in Figure 4, where (a) represents the background image set as a town’s sky, and (b,c) are the scene images at moments $t_{1}$ and $t_{2}$, respectively. There are four moving targets in the scene, denoted as $T_{1},T_{2},T_{3},T_{4}$. The shape of $T_{1}$ and $T_{2}$ is a circle, and the shape of $T_{3}$ and $T_{4}$ is a pentagram. The scene image is 512×512 pixels in size, and the target position at the moment $t_{1}$ is set to $T_{1}\left(t_{1}\right)=\left(20,20\right),T_{2}\left(t_{1}\right)=\left(20,200\right),T_{3}\left(t_{1}\right)=\left(100,200\right),T_{4}\left(t_{1}\right)=\left(200,300\right)$. We assume the moment $t_{1}$ as the initial moment of discovering the targets and estimate the displacement $\left(\Delta x_{i},\Delta y_{i}\right)$ of each target from moment $t_{1}$ to $t_{2}$. The displacements of the four targets are preset to (5,5),(20,0),(−10,20),(20,−10). A Gaussian white noise with $\left(\mu =0,\sigma^{2}=0.001\right)$ is added to the scene image.

#### 3.1.1. Multi-Target Locating

**Figure 5:**
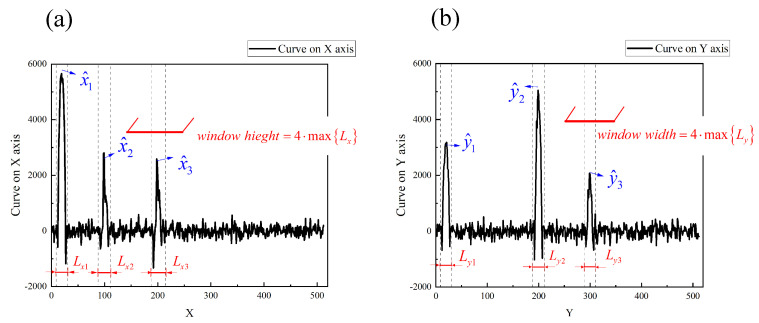
Projected curves of the image with four targets. (**a**) is the projected curve of image scene on *X* axis and (**b**) is the projected curve on *Y* axis. $\left(\hat{x_{1}},\hat{x_{2}},\hat{x_{3}}\right)$ are the three regions on the *X* axis where the target may exist, and $\left(\hat{y_{1}},\hat{y_{2}},\hat{y_{3}}\right)$ are the three regions on the *Y* axis. The length $L_{x}$ and $L_{y}$ of the regions determine the size of window function.

The first experiment located the targets in the scene at moment $t_{1}$, as shown in Figure 4b. The projected curves of the entire target image $I(x,y;t_{1})$ under 2×0.25×512 modulated patterns are shown in Figure 5. These illumination patterns are constructed from a Hadamard matrix according to the method in [23]. The reconstruction method of projected curves is not described in the specific process here.

From the projected curve on the *X* axis shown in Figure 5a, we can determine the four target positions in three regions on the *X* axis. The coordinates of the center points of these three regions on the *X* axis are $\hat{x_{1}}=19$, $\hat{x_{2}}=98$, and $\hat{x_{3}}=198$. Similarly, the center points of the four regions on the *Y*-axis are denoted as $\hat{y_{1}}=20$, $\hat{y_{2}}=199$, and $\hat{y_{3}}=299$. These coordinates $\left(\hat{x},\hat{y}\right)$ define nine regions where targets may exist, that is, nine rectangular window functions. In addition, the size of the spatial window function can be determined by the length $L_{x}$ and $L_{y}$ of the regions in the projected curves. Here we take four times the length of the longest region on the projected curve as the window length of the window function in the *X* or *Y* axis. The size of window function is defined as 64×64 pixels, since the maximum of $\left(L_{x},L_{y}\right)$ is (16,16) pixels.

The 18 Fourier coefficients are calculated corresponding to these nine rectangular window functions are calculated according to Equation (1) and (2). Thus, we can exclude the regions where no targets existed and obtain the exact locations of the centers of four targets, as shown in Table 1.

#### 3.1.2. Multi-Target Tracking Using the Independent Estimation Approach

**Figure 6:**
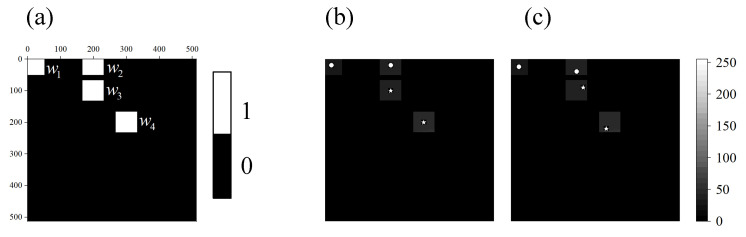
The four window functions for four targets: (**a**) is the four-window function, (**b**) is the windowed image at moment $t_{1}$, and (**c**) is the windowed image at moment $t_{2}$.

**Figure 7:**
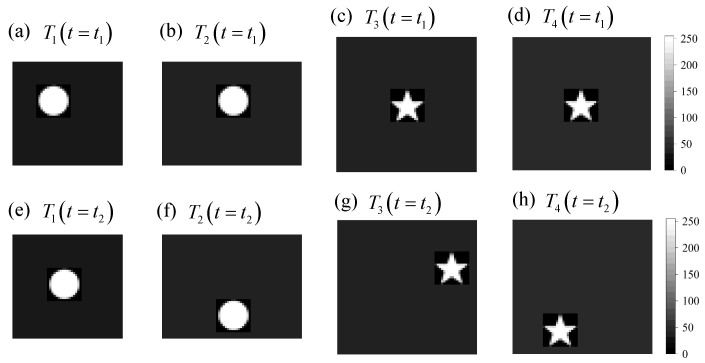
The four window functions: (**a**–**d**) are the images at the moment $t_{1}$, and (**e**–**h**) are the images at the moment $t_{2}$; (**a**,**e**) represent target $T_{1}$, (**b**,**f**) represent target $T_{2}$, (**c**,**g**) represent target $T_{3}$, and (**d**,**h**) represent target $T_{4}$.

The four window functions designed based on the multi-target position are shown in Figure 6. They are centered on ${\bar{w}}_{1}=\left(19,20\right)$, ${\bar{w}}_{2}=\left(20,199\right)$, ${\bar{w}}_{3}=\left(98,199\right)$, and ${\bar{w}}_{4}=\left(198,299\right)$. The parts of $w_{1}$ and $w_{2}$ that are beyond the boundary are discarded. The spatial windows designed for different targets are shown in Figure 7, where (a–d) represent the images at the moment $t_{1}$, and (e–h) represent the images at the moment $t_{2}$. (a,e) represent target $T_{1}$, (b,f) represent target $T_{2}$, (c,g) represent target $T_{3}$, and (d,h) represent target $T_{4}$.

The estimated results of the four targets according to Equation (12) are shown in Table 2. The errors of estimated results are within two pixels. The errors of $T_{3}$ and $T_{4}$ are larger than those of $T_{1}$ and $T_{2}$ because the target $T_{3}$ and target $T_{4}$ have a lower brightness and are more affected by noise.

#### 3.1.3. Multi-Target Tracking Using the Joint Estimation Approach

**Figure 8:**
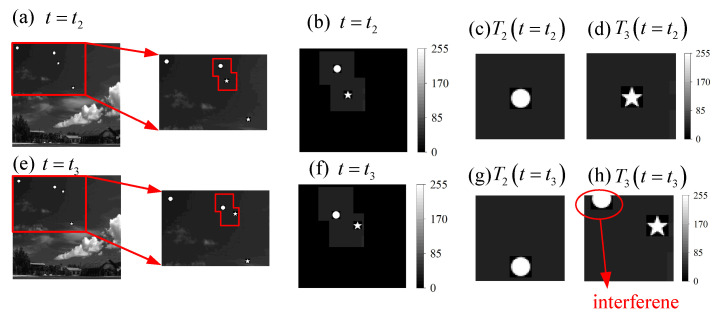
Illustrations of the windowed image at moment $t_{2}$ and $t_{3}$: (**a**–**d**) represent the images at moment $t_{2}$, and (**e**–**h**) represent the images at moment $t_{3}$, (**c**,**g**) are the images of target $T_{2}$, and (**d**,**h**) are the images of target $T_{3}$. (**h**) contains the interference caused by target $T_{2}$.

Assuming that the speed of the four targets remains unchanged, the scene image at the moment $t_{3}$ is as shown in Figure 8. At this moment, target $T_{2}$ and target $T_{3}$ are already very close. By updating the center coordinates of the window functions with the result estimated at the moment of $t_{2}$, we can obtain the results of the window function selected for target $T_{2}$ and target $T_{3}$ at the moment of $t_{2}$ and $t_{3}$, as shown in Figure 8, where (a–d) represent the images at moment $t_{2}$, (e–h) represent the images at moment $t_{3}$, (c,g) are the images of target $T_{2}$, and (d,h) are the images of target $T_{3}$.

Significant errors will occur when estimating the displacement of target $T_{3}$ using the independent estimation method. Using the previously mentioned joint estimation method, the four Fourier coefficients of the windowed target image are calculated at moment $t_{2}$, and the displacement of target $T_{3}$ is solved using the analytical method. The results of the two estimation approaches are shown in Table 3.

The results of $T_{2}$ using the different estimation approach are similar. However, there is a significant error in the result of $T_{3}$ using the independent estimation approach. It is obvious that there is an interference in the target estimation as shown in Figure 8h.

As the number of targets becomes larger, the computational effort of the joint estimation approach increases significantly. In conclusion, the joint estimation approach is mainly used when multiple targets are close. The independent estimation approach is preferred when the distance between different targets is long.

### 3.2. Experiment

**Figure 9:**
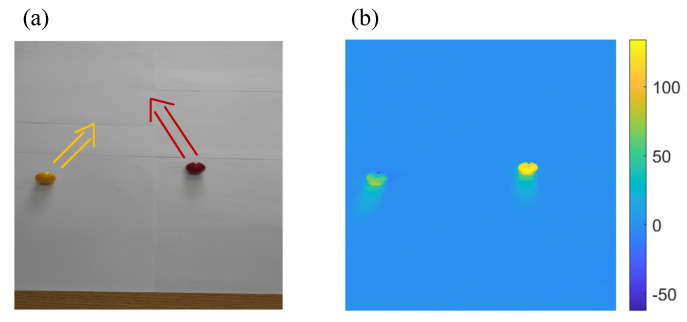
Experiment scene: (**a**) is the 240th frame in the video, and (**b**) is the image of 240th frame with background subtraction.

In order to verify the capability of the proposed method, a video experiment was performed. Two balls representing two targets are moving irregularly in the scene at the same time, as shown in Figure 9. The experiment captured a video through a cell phone camera and performed target tracking for each image frame in the video. The matrix representing the scene brightness distribution of each frame was multiplied by a different sampling matrix, and the sum of the products was assumed to be the detected light intensity of the single-pixel detector. The video size is 1024×720 pixels and was scaled to 512×512 pixels. There are 636 frames in total, and the video frame rate is 60 Hz. The video was shot at a distance of 60 cm at an angle of 45 degrees. The target scene size is 50 cm × 35 cm, and the diameters of the balls are both 1.5 cm.

**Figure 10:**
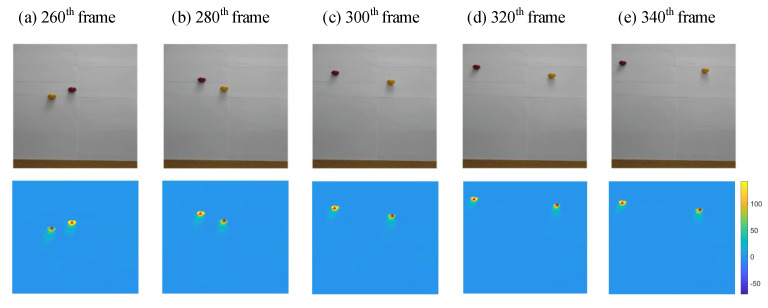
Estimated results in five different frames from the 260th frame to the 340th frame. The first row shows the scene images, and the second row shows the results of multi-target tracking. The real-time estimated position of Target 1 (representing the yellow ball on the left in the 240th frame) is marked with a red dot, and Target 2 (representing the red ball on the right in the 240th frame) is marked with a red triangle.

We assume that the 240th frame is the moment when the targets are first detected. Multi-target tracking was performed for a total of 391 frames from the 240th frame to the 630th frame. Estimated results in five different frames are shown Figure 10 from the 260th frame to the 340th frame. The five frames present the motion of the balls and the estimated results. The first row of the Figure 10 shows the scene images, and the second row shows the results of multi-target tracking. The real-time estimated position of target $T_{1}$ (representing the yellow ball on the left in the 240th frame) is marked with a red dot in Figure 10, and target $T_{2}$ (representing the red ball on the right in the 240th frame) is marked with a red triangle.

As shown in Figure 10, the estimated results and the actual positions of the targets always overlap with small error. It can be concluded from each frame that the estimation error of the proposed multi-target tracking method is less than the radius of the ball. Considering that the ball occupies about 30 pixels in the image, the estimation error is less than 15 pixels. If we manually calibrate these five frames, the average estimation error is about 6 pixels.

**Figure 11:**
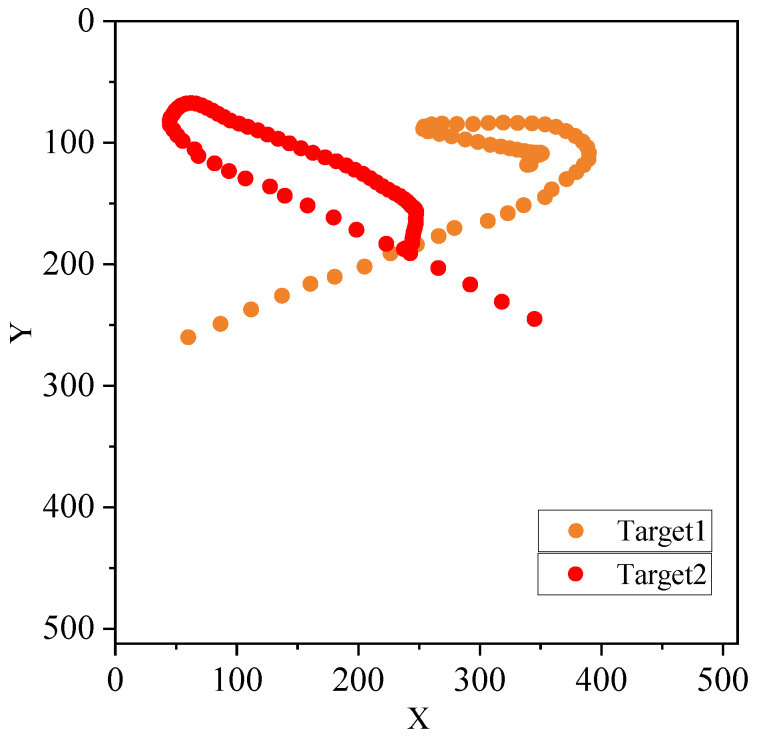
The estimated trajectories.

The estimated trajectories of the two balls are shown in Figure 11. The yellow points are the representation of the left yellow ball in Figure 9a, and the red points represent the right red ball. The trajectories are displayed every five frames.

**Figure 12:**
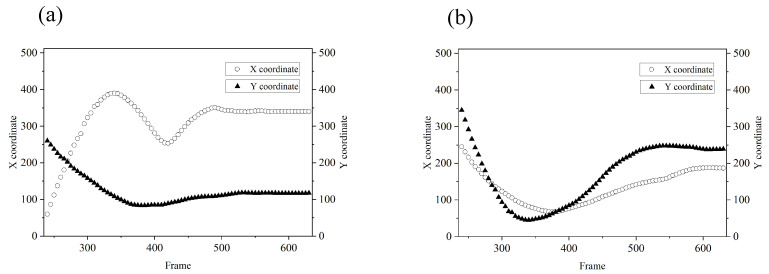
The estimated results of coordinates between the 240th frame and the 630th frame: (**a**) is the yellow ball, and (**b**) is the red ball.

The positions of the yellow ball are shown in Figure 12a every two frames, and the red ball is represented in Figure 12b. Between the 240th frame and the 260th frame, the independent estimation approach was used to track the targets. After the 260th frame, the two targets are close and cannot be distinguished by the spatial window function. The joint estimation approach was used from the 260th frame to the 320th frame. After the 320th frame, the two balls are separated again, and the independent estimation approach was used after the 320th frame.

## 4. Discussion

The shape of the window function affects the tracking performance of the proposed method. In this paper, a common rectangular window function was chosen, and the window length was also preset to be a constant. However, in a practical application, the shape and window length of the window function should be related to the target shape, the target motion speed, and other factors. A large window length is required for high-speed targets, and a small window length is required for multi-target estimation. Therefore, a window function with an adjustable shape will be better suited for this method.

In the joint estimation approach, only the estimation of two targets is shown in this paper. However, theoretically, this method can be used to estimate more than two targets simultaneously. Fewer than five targets can be estimated directly according to the analytical method. When used for more than five targets, the time consumption of the search method is more demanding. Subsequent work will further extend the proposed joint estimation approach for more targets.

## 5. Conclusions

In conclusion, this paper presents a multi-target tracking method using windowed Fourier single-pixel imaging. The proposed method can track *K* targets by implementing 6K measurements using a single-pixel detector. For example, using a DMD with a modulate rate of 10 kHz, the tracking frame rate for five targets was up to 333 Hz. The ability to track multiple targets will greatly broaden the application range of SPI in security and military fields. In particular, a combination with millimeter waveband detectors will further expand SPI’s application range due to the reconfigurable antenna patterns.

## Figures and Tables

**Table 1:** The estimated results of multi-target locations.

Targets	$T_{1}$	$T_{2}$	$T_{3}$	$T_{4}$
Position coordinate	(20,20)	(20,200)	(100,200)	(200,300)
Estimation result	(19,20)	(20,199)	(98,199)	(198,299)

**Table 2:** The results using the independent estimation approach.

Targets	$T_{1}$	$T_{2}$	$T_{3}$	$T_{4}$
True value	(5,5)	(20,0)	(−10,20)	(20,−10)
Estimation result	(4.82,4.60)	(19.67,0.08)	(−8.89,19.65)	(21.15,−8.55)
Error	0.43	0.34	1.16	1.85

**Table 3:** The results using the joint estimation approach.

	Independent Estimation	Joint Estimation	True Value
T2	(19.66, −0.14)	(19.48, 0.15)	(20, 0)
Error of T2	0.37	0.29	
T3	(−23.34, −6.92)	(−11.29, 19.82)	(−10, 20)
Error of T3	30.04	1.30	

## Data Availability

Not applicable.

## Abbreviations

The following abbreviations are used in this manuscript:

SPI	single-pixel imaging
FSI	Fourier single-pixel imaging
WFSI	windowed Fourier single-pixel imaging
STFT	short-time Fourier transform
DMD	digital modulating devices
